# Supplementary material for: Chemical and entropic control on the molecular self-assembly process
Source: Nat Commun. 2017 Feb 14;8:14463. doi: 10.1038/ncomms14463 (PMC5316874; doi:10.1038/ncomms14463)
Supplement: Supplementary Information — Supplementary Figures, Supplementary Tables, Supplementary Notes and Supplementary References. [file ncomms14463-s1.pdf]

Supplementary Note 1. Selection of molecule adsorption conformation

The following procedure was used to choose realistic conformations for the Br_2BA $(NH_2)_2BA$ and Me_2BA molecules when adsorbed to a Cu(111) surface.

Step 1. A gas-phase geometry optimization of the molecule was performed using density functional theory (DFT).

Step 2. A pristine Cu(111) slab was created and the molecule geometry in (*i*) was again optimized with DFT after being placed in the vicinity of the surface. The Cu(111) surface was kept frozen during this step, and no care was taken on exactly where the molecule was placed on the surface.

Step 3. A single unit cell (the 'sample cell') was then selected from the Cu(111) surface and discretized into a grid of 25 points labeled as 1, 2, .., 25 (Supplementary Figure 1). 10 of these points were chosen at random, and for each grid point the molecule conformation from Step 2 was placed on the sample cell of that its center of mass lay directly above the grid point. An orientation of either 0°, 30°, 60°, 90°, 120°, or 150° was assigned at random to the molecule (Supplementary Figure 1). At this stage, the 'unstandardized' orientations were used, i.e., the molecular orientation corresponding to 0° was not specifically set relative to the Cu(111) atomic planes. Moreover, because the molecular conformation from (*ii*) approximately possesses two-fold rotational symmetry, angles between 180° and 360° were not considered.

Step 4. For each of the ten adsorbed molecules generated in Step 3, the molecule conformation was again optimized with DFT, with the surface atoms kept frozen. This calculation therefore produced a random sample of ten adsorption conformations for the molecule on the surface.

Step 5. For each of these ten structures, the distance moved by each atom during the optimization in Step 4 was calculated. Supplementary Table 1 shows the standard deviation of the distances moved by the atoms during this procedure. Cases for which the standard deviation is large (e.g., (NH_2) $_2BA$) indicate that the adsorption configuration is very sensitive to where the molecule is placed on the surface The distances moved by the atoms were averaged over all ten conformations and then added to the coordinates of the atoms in the conformation optimized in Step 2. Step 5 therefore

produces an 'averaged' adsorption conformation.

Step 6. Rotational symmetry was then imposed by rotating the averaged conformation from Step 5 through its center of mass and averaging the positions of the equivalent atoms. Supplementary Table 2 shows the standard deviation of the distance moved by the atoms following this step. The standard deviations in Supplementary Table 2 are very small compared to the values from Supplementary Table 1, indicating that the various adsorption conformations for the molecules are close to being rotationally symmetric. This supports the imposition of rotational symmetry on the molecule conformation in this study.

The adsorption conformations determined by this procedure are shown in Supplementary Figure 2. This procedure produces only one of two chiralities for the adsorbed conformation; the calculations in this paper only employed the chirality shown in Supplementary Figure 2, which is sufficient for characterizing the role of chemical and entropic controls on the molecular self-assembly process.

All DFT calculations described above were performed in VASP 5.3.5 (Supplementary Reference 1). The calculations in step 1 were performed with the PBE exchange-correlation functional (Supplementary Reference 2), whereas the calculations in steps 2 and 3 were performed with the rev-vdW-DF2 exchange correlation functional (Supplementary References 3, 4, 5). All calculations used PAW-PBE pseudopotentials, an energy cut-off of 400 eV, and a 2 x 2 x 1 Monkhorst-Pack *k*-points grid. All Cu(111) slabs consisted of 3 layers of Cu atoms.

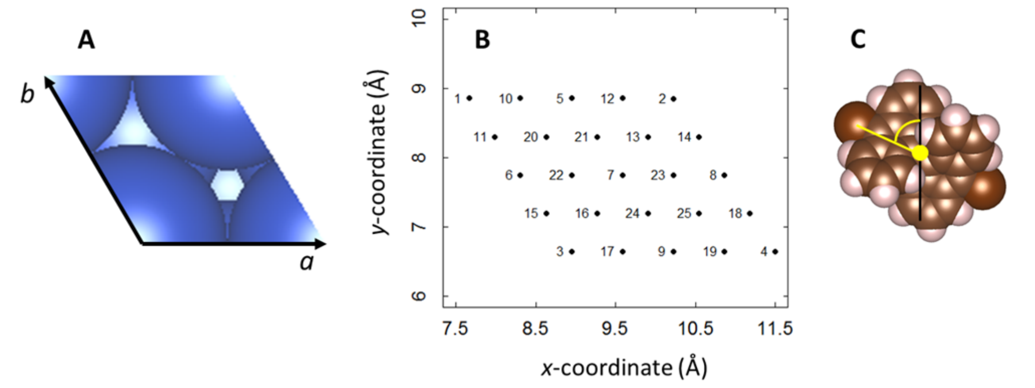

Supplementary Figure 1. (A) A single unit cell from the Cu(111) surface. (B) Discretization of the unit cell into 25 grid points. The ordering of the point labels is due to the computational procedure used to generate the discretization and does not have any physical meaning. (C) Diagram showing how 'orientation' is defined for bianthryl molecules. The yellow point represents the center of mass, and the yellow line coincides with the C-C bond connecting the two anthryl units. Orientation is measured relative to the black line (60° in the case shown). The center-of-mass of the molecule lies directly above one of the points in the middle figure when the molecule is placed on the surface. The direction of the black line was always perpendicular to the *a* axis of the unit cell shown on the left-hand side (expect in the 'unstandardized' calculations of step 3 in Section 1, in which the black line was never calibrated with respect to the Cu(111) lattice planes).

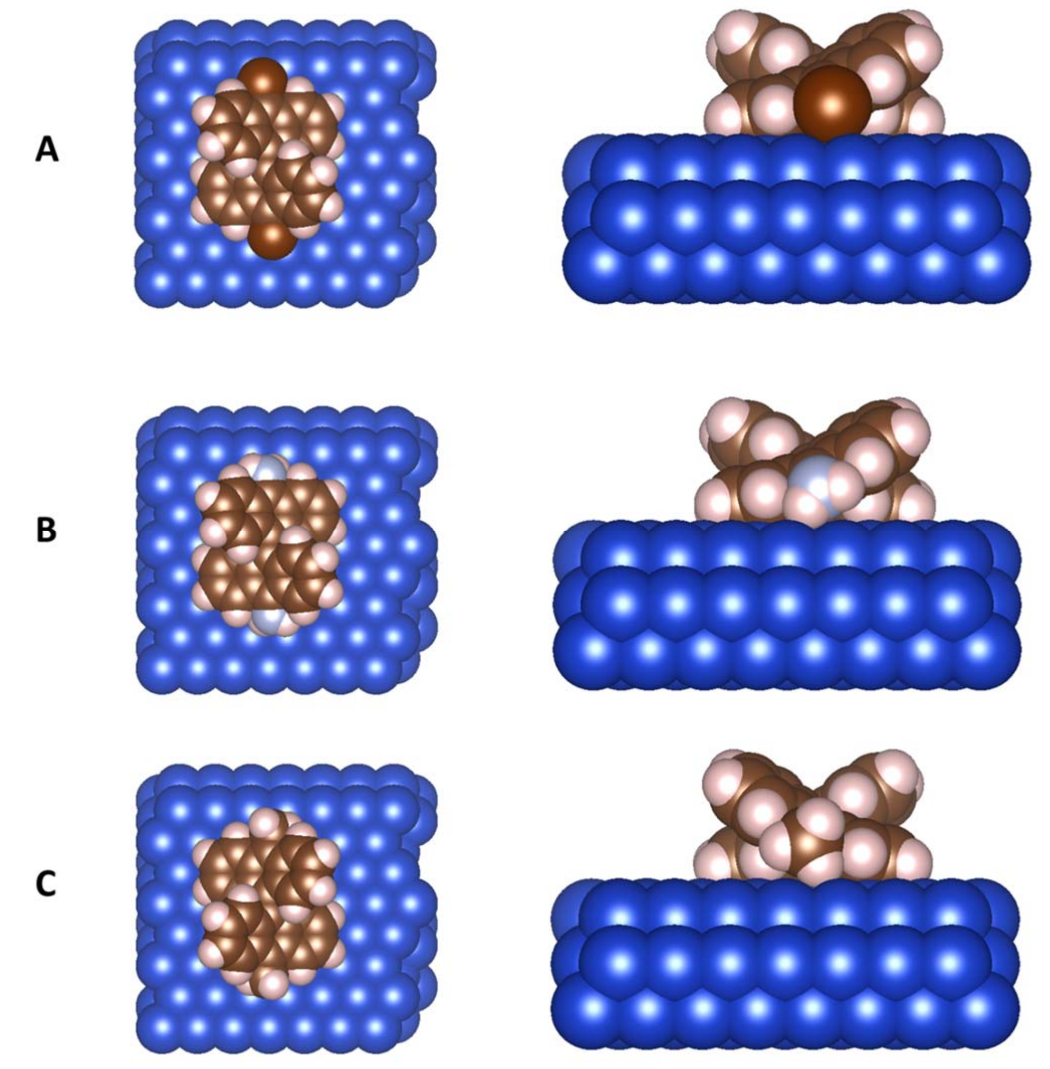

Supplementary Figure 2. Adsorption conformations for Br_2BA (top), $(NH_2)_2BA$ (middle), and Me_2BA (bottom) molecules on Cu(111) surfaces, as determined by the procedure in Section 1. Each molecule is in the 0° orientation. The orientation is standardized to that the C-C bond connecting the two anthryl units is perpendicular to the *a* axis of the Cu(111) unit cell (see Supplementary Figure 1)

	x-coordinate (10^3Å)	y-coordinate (10^3Å)	z-coordinate (10^3Å)
Br_2BA	36.30	32.60	53.20
$(NH_2)_2BA$	5443	5520	41.98
Me_2BA	129.4	171.7	306.1

Supplementary Table 1. Standard deviation of the distances moved by the atoms during Step 5 of the molecule adsorption conformation selection procedure. These numbers measure the variation in the molecule conformations when adsorbed at different parts of the Cu(111) unit cell.

	x-coordinate (Å)	y-coordinate (Å)	z-coordinate (Å)
Br_2BA	5.42	5.64	0.13
$(NH_2)_2BA$	3.64	3.02	0.03
Me_2BA	6.49	4.88	< 0.01

Supplementary Table 2. Standard deviation of the distances moved by the atoms following Step 6 of the molecule adsorption conformation selection procedure. These numbers measure the lack of rotational symmetry of the various molecule adsorption conformations Cu(111) unit cell.

Supplementary Note 2.
Determination of colors, shades, and the adsorption energy function

In the GBA model, a single molecule adsorbate is represented by the combination of one cell, one color, and one shade (Figure 2 of the main paper). The cell and color corresponds to the unit cell and adsorption site in the unit cell, respectively, over which the center of mass of the adsorbed molecule sits. The shade corresponds to the orientation of the adsorbed molecule, as described in the previous section. Colors and shades for the GBA model were determined by identifying the low-energy adsorption sites using DFT calculations. This procedure ran as follows.

Step 1. Using the adsorption conformation for the molecules determined from the procedure in the previous section, the adsorption energy was calculated using DFT for each of the 25 grid points and 6 orientations (0°, 30°, 60°, 90°, 120°, and 150°) shown in Supplementary Figure 1. However, in this case the orientation of the molecule was ‘standardized’ so that the molecular orientation corresponding to the 0° case was fixed relative to the Cu(111) lattice planes (Supplementary Figure 1). In these DFT calculations, all atoms in the molecule and surface were kept static. The adsorption energies for each grid point and orientation (‘adsorption energy maps’) are shown in Supplementary Figure 3 – 5.

Step 2. ‘Low-energy adsorption sites’ were identified as the adsorption states lying in the low-energy ‘trenches’ of in the adsorption energy maps in Supplementary Figure 3 – 5. In Supplementary Figure 3 – 5, these low energy trenches include the grid points labelled as 6, 22, 7, 23, 8 (for the 0° orientation), 5, 20, 6, 8, 25, 9 (for the 60° orientation), and 5, 21, 7, 24, 9 (for the 120° orientation). This procedure identifies nine colors (corresponding the grid points 5, 20, 21, 6, 22, 7, 23, 24 and 25 in Supplementary Figure 3 – 5 where grid points 9 and 5, as well as 6 and 8, are identical by symmetry), where each color has one or two shades (corresponding to the low energy orientations 0°, 60°, or 120°) for the GBA model (Supplementary Figure 6, 7). The same colors and shades were used for each of the three molecules, due to topographically identical adsorption energy maps for each case.

The DFT calculations in step (*i*) were performed in VASP 5.3.5 (Supplementary Reference 1) using PAW-PBE pseudopotentials, the rev-vdW-DF2 exchange correlation

functional (Supplementary Reference 3, 4, 5), an energy cut-off of 400 eV, and a 2 x 2 x 1 Monkhorst-Pack *k*-points grid. The adsorption energy maps were plotted with the AKIMA package for R 3.2.2 (Supplementary References 6, 7). Cu(111) slabs consisting of three Cu layers were used in each case.

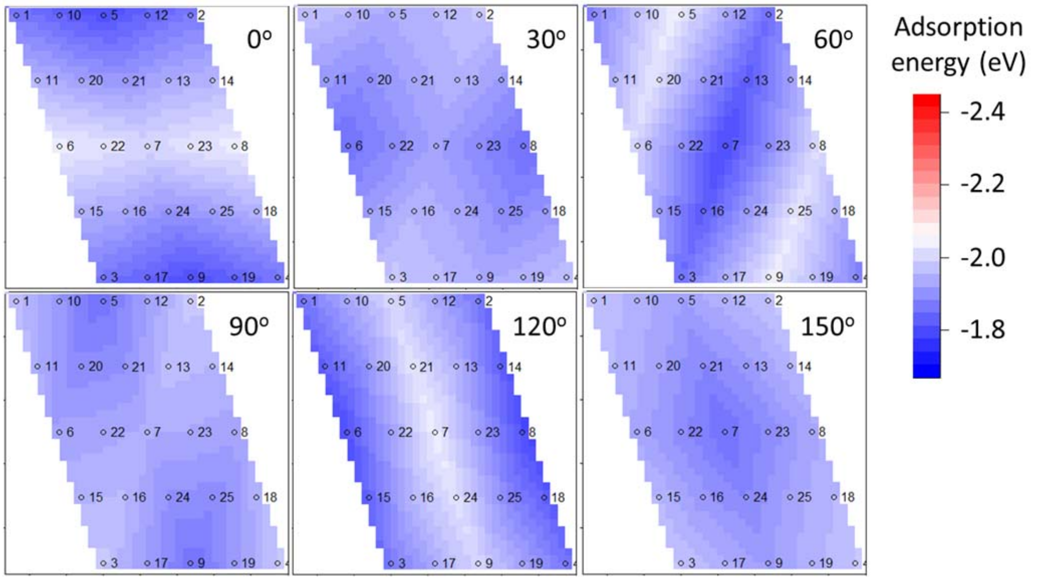

Supplementary Figure 3. Adsorption energy maps for Br_2BA on a Cu(111) unit cell.

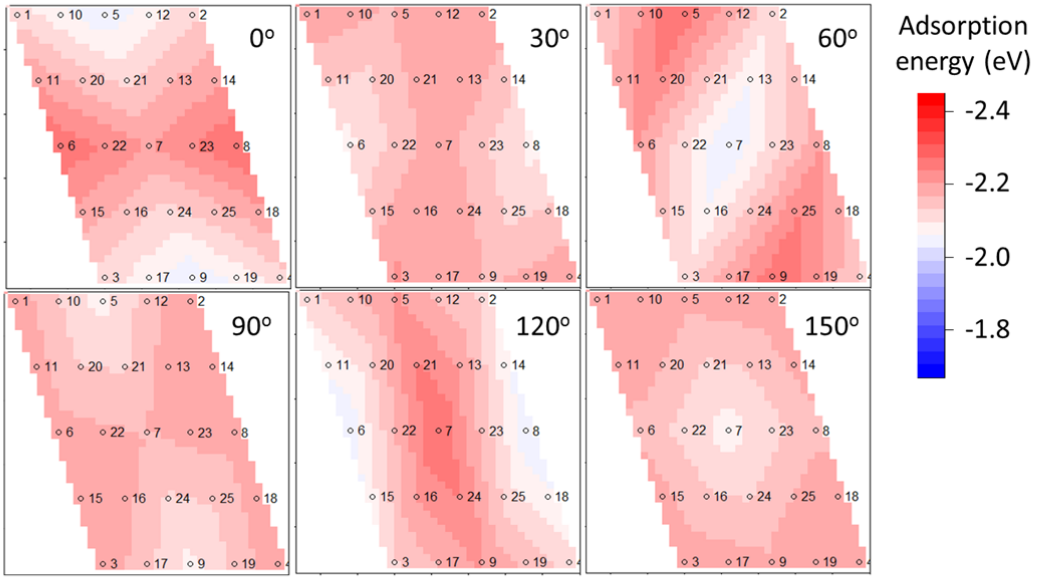

Supplementary Figure 4. Adsorption energy maps for $(NH_2)_2BA$ on a Cu(111) unit cell.

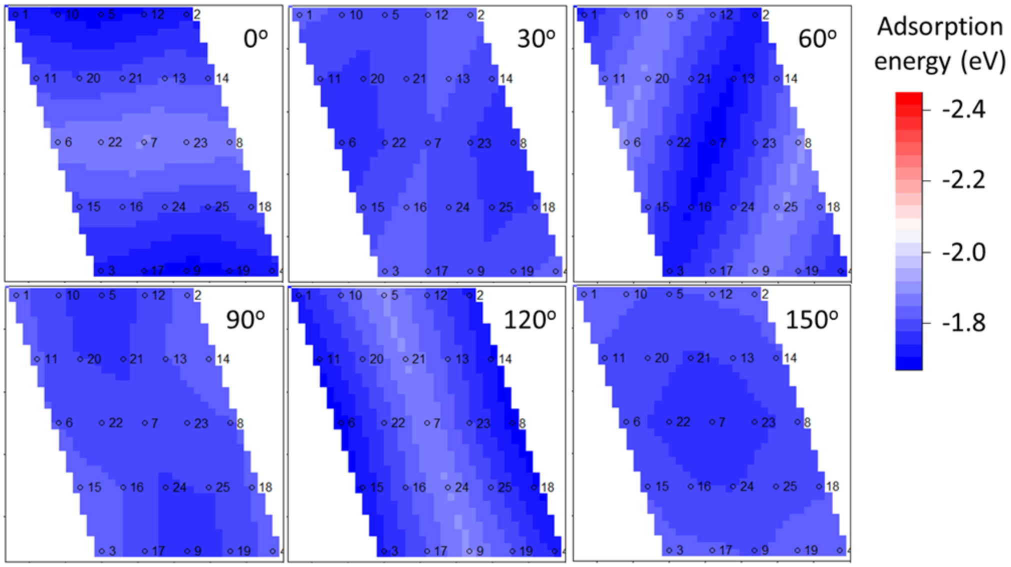

Supplementary Figure 5. Adsorption energy maps for Me_2BA on a Cu(111) unit cell.

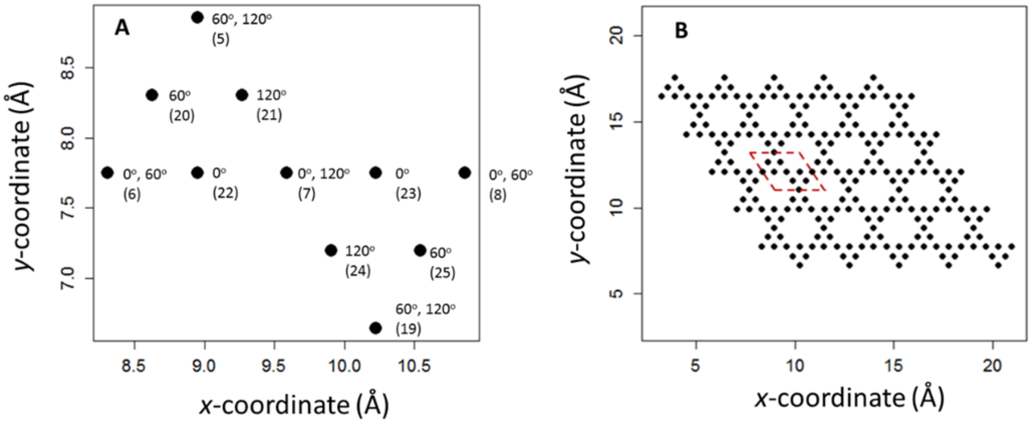

Supplementary Figure 6 (A) Main adsorption sites and orientations for a bianthracene molecule residing over a single Cu(111) unit cell, as determined from the low-energy regions of the adsorption energy maps in Supplementary Figure 3 – 5. This corresponds to a set of colors and shades for the GBA model. The numbers in parentheses correspond to the point labels for the unit cell discretization in Supplementary Figure 1, 3, 4 and 5. (B) Main adsorption sites for a bianthracene molecule residing on a Cu(111) surface containing 5 x 5 unit cells. A single unit cell is indicated in red. While the GBA model considers a square lattice, the actual energy calculations are performed by mapping the cells and colors to the corresponding adsorption site in a grid of the type shown here. Thus, the symmetry of the GBA model does not directly enter into our calculations (also see Supplementary Note 5).

Supplementary Note 3. Validation of Model Assumptions

The accuracy of the entropic component of our calculations is mathematically guaranteed whenever low-coverage conditions are present (see Supplementary Note 5). On the other hand, the accuracy of the energetic component of our calculations is determined entirely by the energy function (equation (1) of the main text), which makes a number of assumptions on the surface-molecule and molecule-molecule interactions. Given the accuracy of the entropy component of or calculations, it is therefore sufficient to confirm the assumptions of the energy function in order to validate the GAMMA modeling approach.

Provided that the intermolecular interaction energy cut-off is sufficiently large, so that the interaction between islands can be neglected, the energy for configuration *c* can be written as

$$E(c)=\sum_{I\in c}\varepsilon(I) \qquad (1)$$

where $\varepsilon(I)$, the energy of island I, is

$$\varepsilon(I)=\sum_{z\in I}v(z)+\frac{1}{2}\sum_{z_i,z_j\in I}u(z_i,z_j), \qquad (2)$$

where $v(z)$ is the interaction energy between molecule z and the surface, and $u(z_i, z_j)$ is the energy of the interaction between molecules z_i and z_j. Supplementary Equation (2) makes three assumptions.

Assumption 1 The total island-surface interaction energy can be approximated by the sum of the molecule-surface interaction energies in the island.

Assumption 2 The total intermolecular interaction energy can be approximated by the sum of all pairwise intermolecular energies in the island.

Assumption 3 The state of molecule z_j is not affected by the presence of other molecules. ‘State’ means the adsorption location, orientation, and

conformation of the adsorbed molecule.

A more precise statement of Assumption 3 is that $v(z_i)$ and $u(z_i, z_j)$ do not depend upon the other molecules in the island. In addition to the above, the following assumption was also used for calculating the surface-molecule interaction energies.

Assumption 4 The Cu(111) surface can be modelled as a 3-layer slab of Cu atoms.

Note that Assumption 4 is only relevant to the calculation of the quantity $v(z)$, and is independent of Supplementary Equation (2).

Assumptions 1 – 4 were validated *via* DFT calculations performed for the eight islands shown in Supplementary Figure 7. Each island contains three molecules. Consideration of larger islands is difficult because of the prohibitive size increase of the Cu(111) surface. Only two types of Br_2BA islands were considered due to the small variation in shapes for islands containing 3 Br_2BA molecules in our simulations. The DFT calculations were performed in VASP 5.3.5 (Supplementary Reference 1) using PAW-PBE pseudopotentials, the rev-vdW-DF2 exchange correlation functional (Supplementary References 3, 4, 5), an energy cut-off of 400 eV, and a 2 x 2 x 1 Monkhorst-Pack *k*-points grid. Unless mentioned otherwise, a three layer Cu(111) surface and static atoms were considered.

<u>*Validation of Assumption 1*</u>

For each island shown in Supplementary Figure 7, the total island-surface interaction energy $E_{\text{island-surface}}$ was calculated as

$$E_{\text{island-surface}} = E_{\text{total}} - E_{\text{surface}} - 3E_{\text{molecule}}, \qquad (3)$$

where E_{total} is the total energy of the system, E_{surface} is the total energy of the surface alone, and E_{molecule} is the total energy of a single molecule. The total island-surface interaction energy was compared to the approximation

$$E^*_{\text{island-surface}} = \sum_{k=1}^{3} \left(E_{k\text{-surface}} - E_{\text{surface}} - E_{\text{molecule}} \right), \qquad (4)$$

where $E_{k\text{-surface}}$ is the energy of the interaction between molecule k and the surface, in the absence of the other molecules in the island.

Calculations show that $E_{\text{island-surface}}$ and $E^*_{\text{island-surface}}$ compare favorably (Supplementary Table 3), with $E^*_{\text{island-surface}}$ being between about 1.03 to 1.04 times of $E_{\text{island-surface}}$ for the islands studied here. The energy differences $E^*_{\text{island-surface}} - E_{\text{island-surface}}$ are about -0.241 eV ± 0.001 eV (Br_2BA case), -0.225 eV ± 0.085 eV ($(NH_2)_2$BA case), and -0.188 eV ± 0.03 eV (Me_2BA case), where the ±bounds are the difference between the largest and smallest energy differences. The variation in this error may be comparable to some weakly attractive intermolecular interaction within an island, however this variation is not expected to be significant for self-assembly calculations, in which strong attractive interactions are important.

The fact that $E^*_{\text{island-surface}}$ is generally larger than $E_{\text{island-surface}}$ probably indicates that a small 'charge cushion' is present around the base of each adsorbed molecule, due to the displacement of charge from the surface or molecule following the adsorption of the molecule (Supplementary Reference 8). This would induce repulsive interactions between adsorbed molecules. Because these charge cushions should be localized about each molecule, we do not expect for the ratio of $E^*_{\text{island-surface}}$ and $E_{\text{island-surface}}$ to increase significantly for larger islands. In any case, further theoretical work on the origin of these discrepancies would make for a useful future research topic.

Validation of Assumption 2

The total intermolecular interaction energy for each island was calculated as

$$E_{\text{interaction}} = E_{\text{island}} - 3E_{\text{molecule}}, \quad (5)$$

where E_{island} is the energy of the island in the absence of the surface. This was compared to

$$E^*_{\text{interaction}} = \sum_{i,j=1,i\neq j}^{3} \left(E_{ij} - 2E_{\text{molecule}}\right), \quad (6)$$

where E_{ij} is the total energy of the two molecules i and j from the island, in the absence of the third molecule and the surface.

Supplementary Table 3 shows that $E^*_{interaction}$ and $E_{interaction}$ compare well, with $E^*_{interaction}$ being a fraction of about 0.97 to 1.03 of $E_{interaction}$. Moreover, the energy differences $E^*_{interaction}$ -$E_{interaction}$ are between 0.001 eV to 0.015 eV and are negligibly small. Thus, third-order contributions to the total intermolecular interaction energy are negligible for the island studied here. Higher order contributions to the intermolecular interaction energy for larger islands are also expected to be negligible.

Validation of Assumption 3

To test Assumption 3, we attempted to relax the islands structures shown in Supplementary Figure 8 using the same DFT settings described above. Each of these islands appeared in the Monte Carlo simulation output discussed in the main paper, and are therefore predicted to be energetically stable. In particular, each molecule is adsorbed at one of the sites shown in Supplementary Figure 6. These islands are particularly relevant for testing Assumption 3, because they include both 'chain-like' bianthryl tip interactions and interactions between functional groups. These types of interactions feature prominently in the islands predicted by our calculations. To relax these islands, conjugate gradient relaxation was applied with a convergence criterion of 0.05 eV/Å for the force. The bottom layer of Cu(111) atoms were kept frozen during the calculations.

The relaxed structures in Supplementary Figure 8 show no remarkable differences from their non-relaxed counterparts, indicating that the islands predicted by our theory are indeed local energetic minima. More generally, they show that the state of the adsorbed molecules are not significantly affected by the presence of other molecules in the island, and hence that the energetic stability of the island are dominated by the molecule-surface interaction.

Validation of Assumption 4

Island-surface interaction energies computed according to Supplementary Equation (3) for the case of a four-layer Cu(111) surface ($E^4_{island\text{-}surface}$) are compared to three-layer Cu(111) surface calculations ($E^3_{island\text{-}surface}$) in Supplementary Table 4. The comparison appears favorable, with $E^3_{island\text{-}surface}$ being between 97% - 98% of $E^4_{island\text{-}surface}$. The energy differences $E^4_{island\text{-}surface} - E^3_{island\text{-}surface}$ derived from Supplementary Table 4 are

about -0.152 eV ± 0.03 eV (Br_2BA case), -0.165 eV ± 0.01 eV ($(NH_2)_2BA$ case), and -0.149 eV ± 0.007 eV (Me_2BA case), where the ±bounds are the difference between the largest and smallest energy differences.

Assuming a 3-layer Cu(111) surface instead of a 4-layer Cu(111) surface is therefore equivalent to adding a error of about 0.15 eV (depending on the molecule) to each island energy with negligible error variation (note that in the calculations reported in the paper, the interaction energies with magnitudes less than 0.03 eV were approximated as zero in the calculating the interaction energy function; see section 4). This constant error would cancel during Monte Carlo simulations.

On the other hand, the calculations above compare *island*-surface interaction energies. However, in the model calculation, the island-surface interaction is approximated by the sum of molecule-surface interaction energies. In order to test the validity of using a 3-layer Cu(111) surface for calculating the energy function parameters of the GBA model, we computed the adsorption energy map for a single Br_2BA molecule on a 4-layer Cu(111) slab (Supplementary Figure 9). No significant qualitative or quantitative differences between the 3-layer and 4-layer Cu(111) calculations were observed. This shows that the sum of molecule-surface interaction energies for any Br_2BA island is insensitive to the choice of a 3-layer or a 4-layer slab. Similar results are expected for $(NH_2)_2BA$ and Me_2BA molecules as well, since the surface-molecule interaction is dominated by epitaxy of the bianthryl unit with the Cu(111) surface lattice planes.

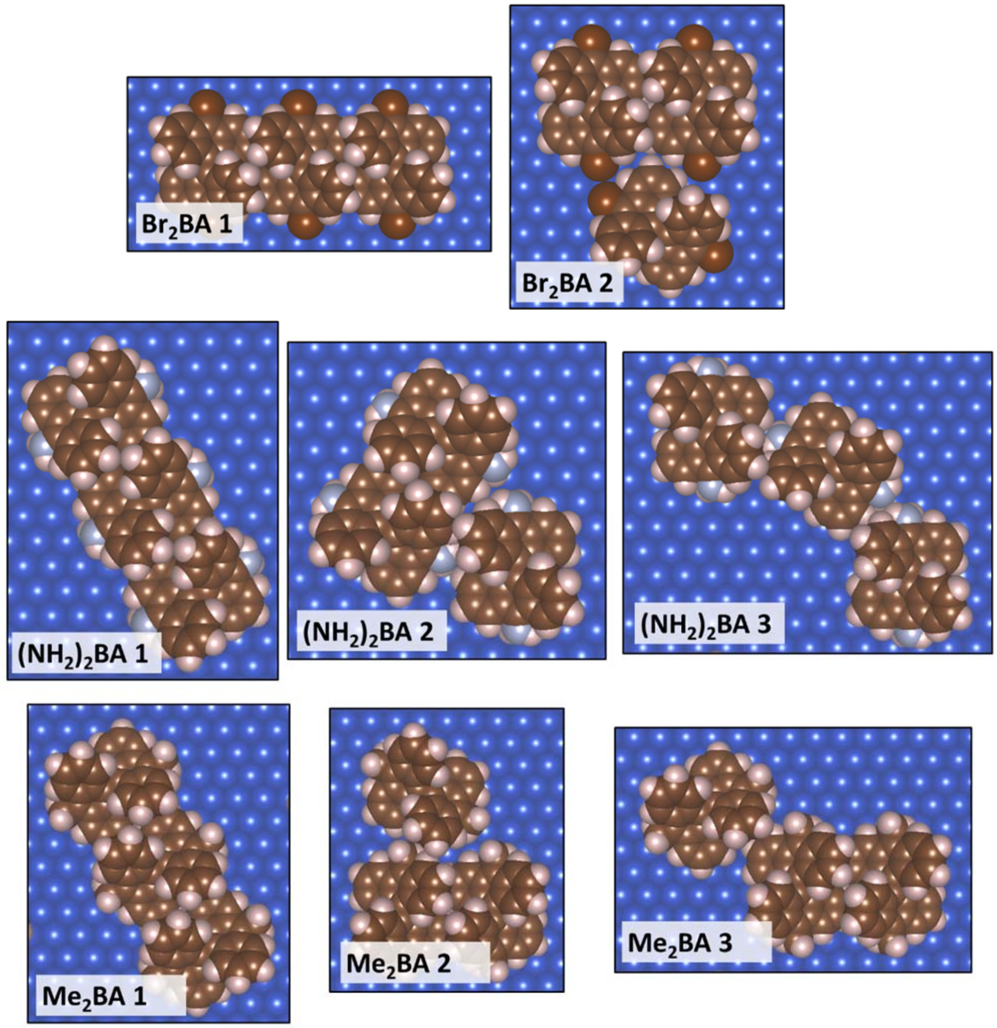

Supplementary Figure 7. Islands used to validate the model energy function.

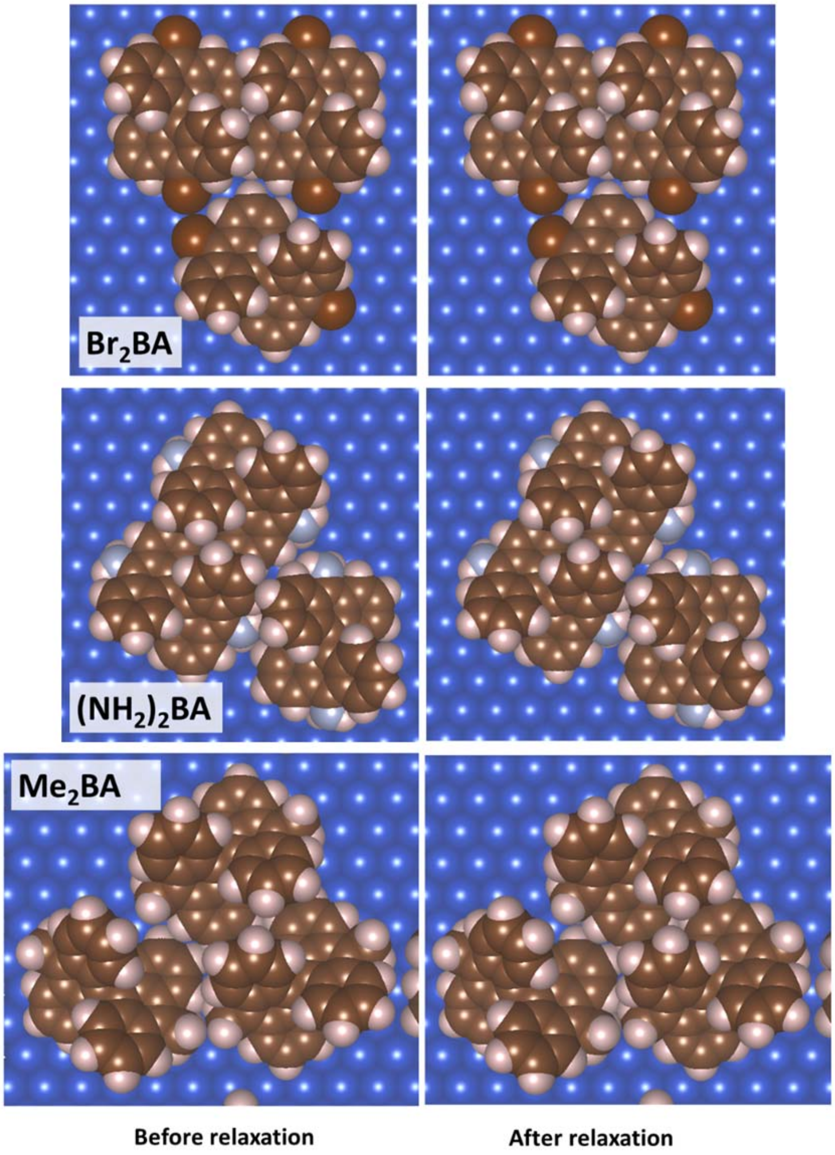

Supplementary Figure 8. Three islands before and after relaxation *via* DFT calculations. Relaxation had no notable effect on the island structures. A small simulation cell was used for the $(CH_3)_2BA$ calculation, and so this island is very close to its images in the neighboring cells.

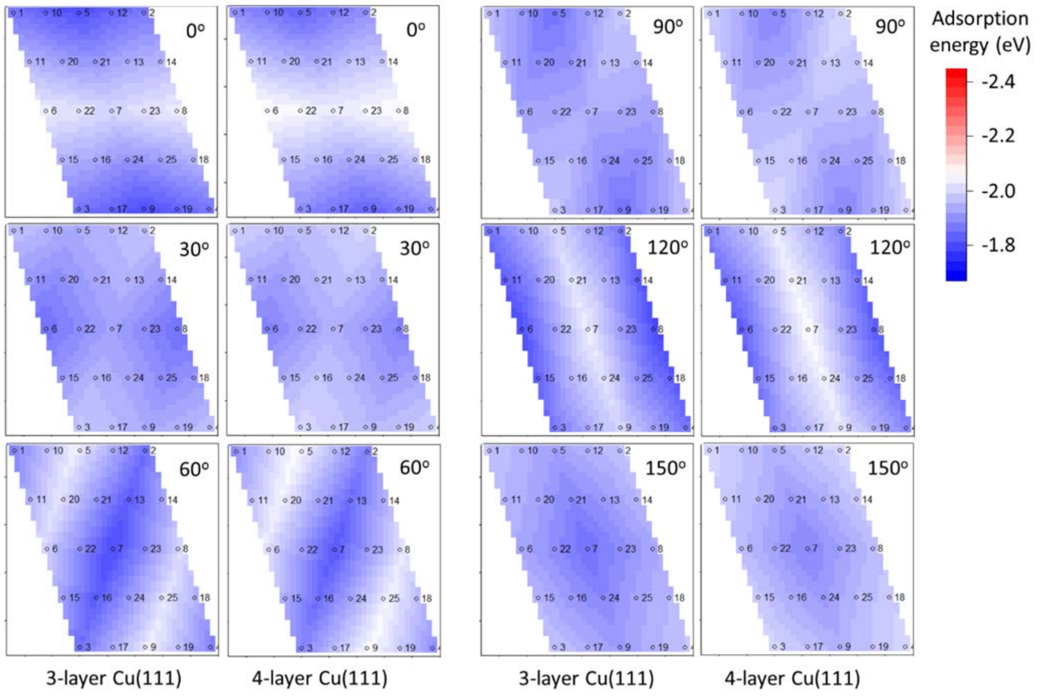

Supplementary Figure 9. Adsorption energy maps for Br_2BA on a the unit cell of a 3-layer Cu(111) slab and a 4-layer Cu(111) slab.

	$E_{\text{island-surface}}$	$E^*_{\text{island-surface}}$	$E^*_{\text{island-surface}}/E_{\text{island-surface}}$
Br_2BA 1	-5.756	-5.997	1.042
Br_2BA 2	-5.578	-5.818	1.043
$(NH_2)_2BA$ 1	-6.459	-6.646	1.029
$(NH_2)_2BA$ 2	-6.065	-6.337	1.045
$(NH_2)_2BA$ 3	-6.009	-6.226	1.036
Me_2BA 1	-5.404	-5.591	1.035
Me_2BA 2	-5.235	-5.438	1.039
Me_2BA 3	-5.365	-5.538	1.032

Supplementary Table 3. Values of $E_{\text{island-surface}}$ and $E^*_{\text{island-surface}}$ computed for the islands shown in Supplementary Figure 7. All energies are in units of eV. See the text surrounding Supplementary Equation (3) and (4) for details.

	$E_{\text{interaction}}$	$E^*_{\text{interaction}}$	$E^*_{\text{interaction}}/E_{\text{interaction}}$
Br_2BA 1	-0.422	-0.407	0.966
Br_2BA 2	-0.379	-0.385	1.017
$(NH_2)_2BA$ 1	-0.436	-0.437	1.001
$(NH_2)_2BA$ 2	-0.309	-0.315	1.021
$(NH_2)_2BA$ 3	-0.115	-0.118	1.029
Me_2BA 1	-0.404	-0.403	0.998
Me_2BA 2	-0.350	-0.347	0.993
Me_2BA 3	-0.285	-0.292	1.024

Supplementary Table 4. Values of $E_{\text{interaction}}$ and $E^*_{\text{interaction}}$ computed for the islands shown in Supplementary Figure 7. All energies are in units of eV. See the text surrounding Supplementary Equation (S5) and (S6) for details.

	$E^3_{\text{island-surface}}$	$E^4_{\text{island-surface}}$	$E^3_{\text{island-surface}}/E^4_{\text{island-surface}}$
Br_2BA 1	-5.756	-5.893	0.977
Br_2BA 2	-5.578	-5.734	0.973
$(NH_2)_2BA$ 1	-6.459	-6.620	0.976
$(NH_2)_2BA$ 2	-6.065	-6.227	0.974
$(NH_2)_2BA$ 3	-6.009	-6.180	0.972
Me_2BA 1	-5.404	-5.549	0.974
Me_2BA 2	-5.235	-5.385	0.972
Me_2BA 3	-5.365	-5.516	0.973

Supplementary Table 5. Values of the island-surface interaction for the 3-layer Cu(111) case ($E^3_{\text{island-surface}}$) and 4-layer Cu(111) case ($E^4_{\text{island-surface}}$) computed for the islands shown in Supplementary Figure 7. All energies are in units of eV. See the text surrounding Supplementary Equation (3) for details.

Supplementary Note 4. Calculation of the Interaction Energy Function

The GBA model considered here employed 50 x 50 = 2500 cells. There are more than 2500 cells x 9 colors x 1 shades = 22500 molecules that can be considered, and hence more than 22500 x 22500 = 5 x 10^8 pairwise molecule-molecule interactions that might occur. Computing the interaction energy *via* DFT for a single molecule-molecule interaction takes around 7 minutes on our computation resources, which implies that over 6000 years are needed to fully compile the interaction energy function $u(z_i, z_j)$. While these arguments do not take symmetry or energy cut-offs into account, a complete tabulation of $u(z_i, z_j)$ is nonetheless unreasonable in the general case. We therefore employ the following machine learning method to estimate $u(z_i, z_j)$. To avoid confusion, 'molecules' in the following refers to the 'real' adsorbate molecules shown in Supplementary Figure 2, rather than the abstract molecules of the GBA model.

Sample generation

Step 1. A random sample of pairwise interactions was generated as follows. Two cell-color-shade combinations were randomly selected, and for each pair two molecules were generated. These molecules had the conformations shown in Supplementary Figure 2, and positions and orientations given according to the cell-color-shade combination. Pairwise interactions that resulted in atom-atom distances between molecules of less than 1 Å were rejected, as were interactions in which the two molecules were separated by a minimum atom-atom distance of more than 8 Å. This procedure resulted in a sample of around 4000 to 5000 random pairwise interactions, denoted as $x_1, x_2, \ldots$.

Step 2. The interaction energy for each pairwise interaction in the sample was calculated *via* DFT. These calculations were performed in the 'gas-phase', i.e., without including the Cu(111) surface. The molecule conformations were also kept static. These calculations were performed in VASP 5.3.5 using a 1 x 1 x 1 gamma-point grid with a 400 eV energy cut-off, PAW-PBE pseudopotentials, and the rev-vdW-DF2 exchange-correlation function (Supplementary References 3, 4, 5).

Feature vectors for the pairwise interactions

In order to employ machine learning techniques, each pairwise interaction must be represented by a *feature vector*, a typically high-dimensional, real-valued vector which encodes the physical content of the interaction. The feature vector used in this study was constructed as follows.

Step 1. Consider a particular pairwise interaction x_i, and let n be the number of atoms in the two molecules. Define the Coulomb matrix $\mathbf{M} = [M_{ij}]_{n \times n}$, where (Supplementary References 9, 10)

$$M_{ij} = \frac{d_i d_j}{\left|\mathbf{r}_i - \mathbf{r}_j\right|}, \qquad (7)$$

where d_i and d_j are the atomic numbers of atom i in molecule 1 and atom j in molecule 2, respectively, and $|\mathbf{r}_i - \mathbf{r}_j|$ is the distance between these two atoms. Here, the molecules are arbitrarily labelled as 1 or 2. The pairwise interaction x_i is then represented by the feature vector

$$x_i = \left(M_{11}, M_{12}, \ldots, M_{n,n-1}, M_{nn}\right). \qquad (8)$$

This representation is unambiguous providing that the atoms of the molecules are labeled and ordered in the same way for all pairwise interactions. This feature vector encodes the Coulombic part of the intermolecular interaction, however a better feature vector might be constructed by explicit consideration of exchange interactions as well.

Step 2. The representation in (S8) is 42 x 42 = 2116 dimensional (for the case of Br_2BA), and so dimensionality reduction was performed with principal component analysis, which typically identified between seven to nine dimensional representations for the pairwise interactions, depending upon the molecule under consideration (Supplementary Figure 10). Principal component analysis was performed with the `prcomp()` routine in R 3.2.2 (Supplementary Reference 6).

<u>*Estimation by machine learning*</u>

We wish to fit the sample data to a function $\hat{u}$ which approximates the true interaction energy function u. However, consider Supplementary Figure 11, which shows a

histogram of the DFT-calculated interaction energies for the sample of pairwise interactions generated in part 3.1 for the case of Br_2BA. The 'repulsive' interactions (those with $u > 0$) span a scale of about 200 eV, whereas the 'attractive' interactions (those with $u \leq 0$) span a much smaller scale of about 0.25 eV. If we perform, say, regression analysis on the sample data, then the resulting function is unlikely to fit well to the attractive interactions because the analysis will not notice these data points. This situation is unacceptable when modeling a phenomenon such as molecular self-assembly, in which attractive intermolecular interactions are a necessary part of the phenomenon. We therefore proceed as follows.

Step 1. Let x be an arbitrary pairwise interaction. From here on, we say that x is an *attractive interaction* if $u(x) < -\varepsilon$, where ε is a small positive constant, a *zero interaction* if $-\varepsilon \leq u(x) \leq \varepsilon$, a *repulsive interaction* if $\varepsilon < u(x) < \varepsilon_c$, where ε_c is a positive constant, and an *unstable interaction* if $\varepsilon_c < u(x)$ (Supplementary Figure 12)

Step 2. We then train a *support vector machine* to classify pairwise interactions as attractive, zero, repulsive, or unstable. Our support vector machine is shown in Supplementary Figure 13 and performance on test data is shown in the caption. This support vector machine was fit using the `svm()` routine from the package `e1071` for R 3.2.2 (Supplementary Reference 11). Gaussian kernels were used, and the optimal parameters were determined by selecting those which performed best against a set of test data.

Step 3. A kernel ridge regression estimator $\hat{g}$ was fit to the subset of sample data corresponding to attractive interactions. This was performed using the routine `krls()` from the package `KRLS` for R. 3.2.2 (Supplementary Reference 13). A separate kernel ridge regression estimator $\hat{G}$ as similarly fit to the subset of sample data corresponding to repulsive interactions, however in this case fitting was performed on the logarithm of the interaction energies. Gaussian kernels were used, and optimal scale parameter was determined by comparison to a set of test data. The regularization parameter is chosen internally in the `krls()` routine by minimization of the sum of squared leave-one-out errors. Performance of the estimators on test data is shown in Supplementary Figure 14.

Step 4. Given a new pairwise interaction x' not present in the original sample, the interaction energy $u(x')$ is estimated as follows. First, the support vector machine trained in step (*ii*) is used to determine whether x' is an attractive, zero, repulsive, or

unstable interaction. Then, we set

$$\hat{u}(x') = \begin{cases} \hat{u}_a(x') = \hat{g}(x') & \text{if } x' \text{ is attractive} \\ 0 & \text{if } x' \text{ is zero} \\ \hat{u}_r(x') = \exp\left(\hat{G}(x')\right) & \text{if } x' \text{ is repulsive} \\ \infty & \text{if } x' \text{ is unstable} \end{cases} \quad (9)$$

where $\hat{g}$ and $\hat{G}$ are the kernel ridge regression estimators fit in step 3.

The explicit treatment of 'zero' interactions and 'unstable interactions' is useful, because these kinds of interactions tend to be over represented in the sample data, which therefore make fitting of the attractive and repulsive kernel ridge regression estimators difficult. We set ε = 0.03 eV and ε_c = 3 eV for each of the molecules considered here. The performance of these estimators on test data is generally satisfactory. A small number of outliers occur when estimating the repulsive energies, however this is acceptable because the repulsive interaction does not need to me modelled so accurately when dealing with molecular self-assembly.

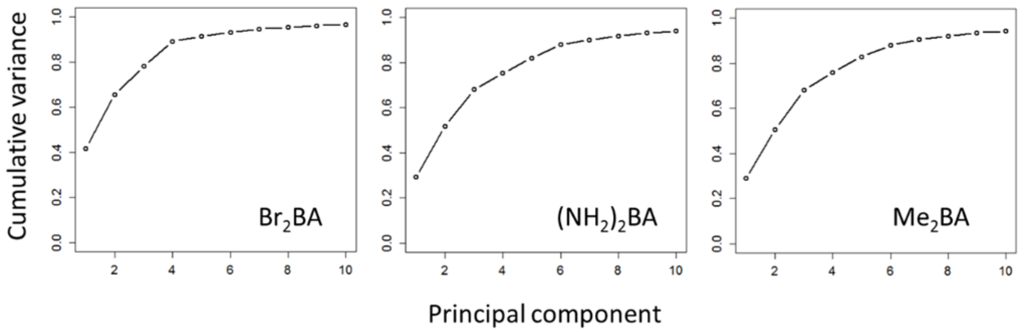

Supplementary Figure 10. Cumulative variance of the first 10 principal components calculated for Br_2BA, $(NH_2)_2BA$, and Me_2BA. The first 7 (respectively, 9, 9) principal components for Br_2BA (respectively, $(NH_2)_2BA$, and Me_2BA) account for 94.6 % (respectively 93 %, 93.5 %) of the total variance of the sample data. Low dimensional vectors consisting of the first 7 (respectively 9, 9) principal components determined above were used in place of the high-dimensional feature vectors in Supplementary Equation (8)

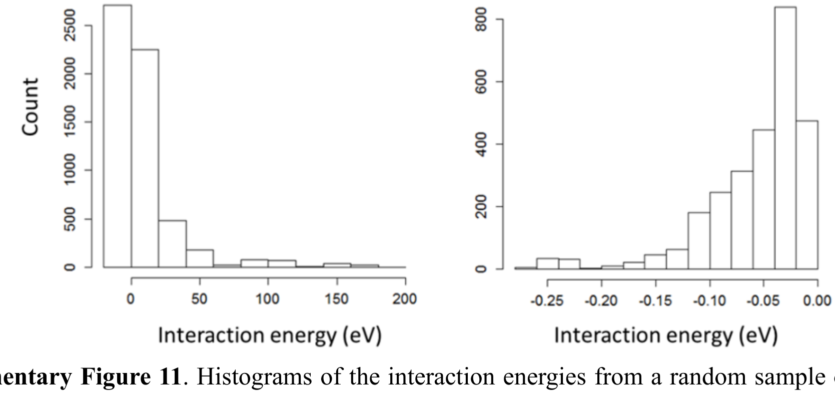

Supplementary Figure 11. Histograms of the interaction energies from a random sample of pairwise interactions (Br_2BA case). The histogram on the left is for the entire sample, and the histogram on the right is for the attractive interactions only. The attractive interactions span a very small range of the entire data set (around 0.25/200 = 0.13 %), and should be treated separately when fitting the energy function.

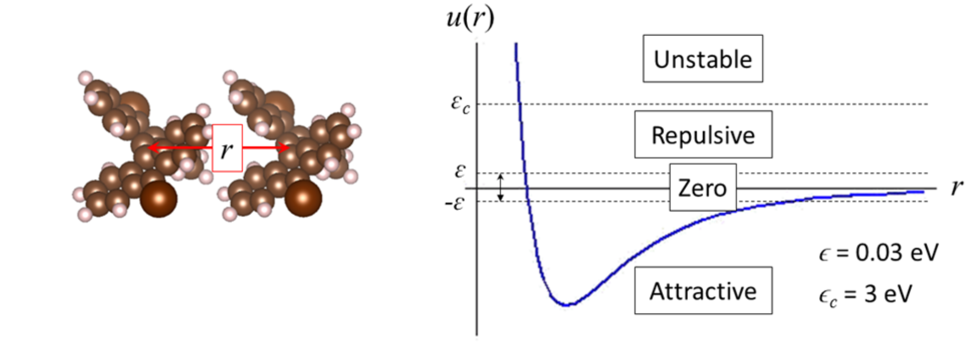

Supplementary Figure 12. Physical interpretation of unstable, repulsive, zero, and attractive interactions. The blue line is a potential curve plotted as a function of the distance between the center-of-masses of the interacting molecules. Potential curve image adapted from a generic plot of an interaction curve (Supplementary Reference 12)

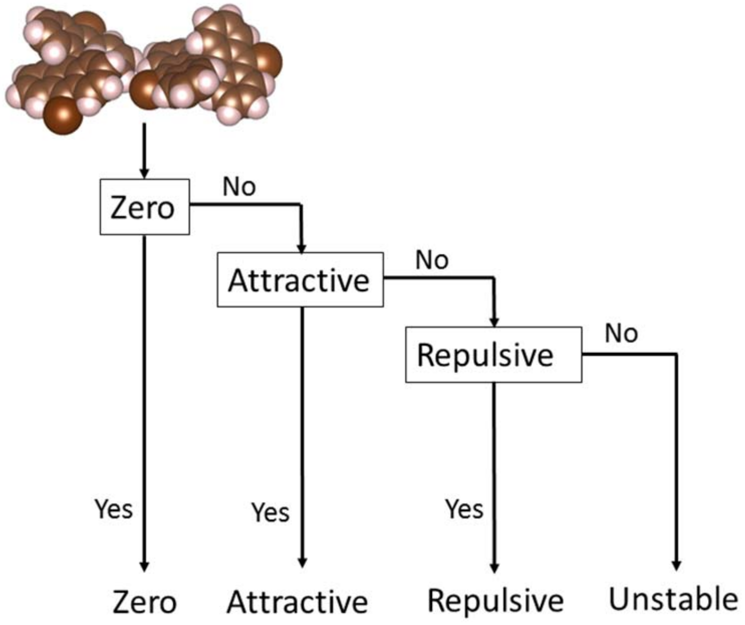

Supplementary Figure 13. Method for classifying a pairwise interaction not belonging to the sample. First, a support vector machine determines if the interaction is in the zero region or not. If not, a second support vector machine determines if the interaction is in the attractive region or not. If not, a third support vector machine determines if the interaction is in the repulsive region or not. If not, the interaction is classified as unstable.

Performance of the first (zero) support vector machine:

Br_2BA: 5252 training points, 582 test points, 1733 support vectors, 7.50 % fail rate on test data

$(NH_2)_2BA$: 4755 training points, 529 test points, 1755 support vectors, 7.37 % fail rate

Me_2BA: 4831 training points, 537 test points, 1957 support vectors, 7.82 % fail rate

Performance of the second (attractive) support vector machine

Br_2BA: 2762 training points, 307 test points, 1266 support vectors, 1.70 % fail rate

$(NH_2)_2BA$: 3369 training points, 375 test points, 1317 support vectors, 1.06 % fail rate

Me_2BA: 4136 training points, 460 test points, 1613 support vectors, 1.09 % fail rate

Performance of the third (repulsive) support vector machine

Br_2BA: 2762 training points, 307 test points, 870 support vectors, 2.00 % fail rate

$(NH_2)_2BA$: 2113 training points, 235 test points, 716 support vectors, 3.40 % fail rate

Me_2BA: 2186 training points, 243 test points, 681 support vectors, 3.29 % fail rate

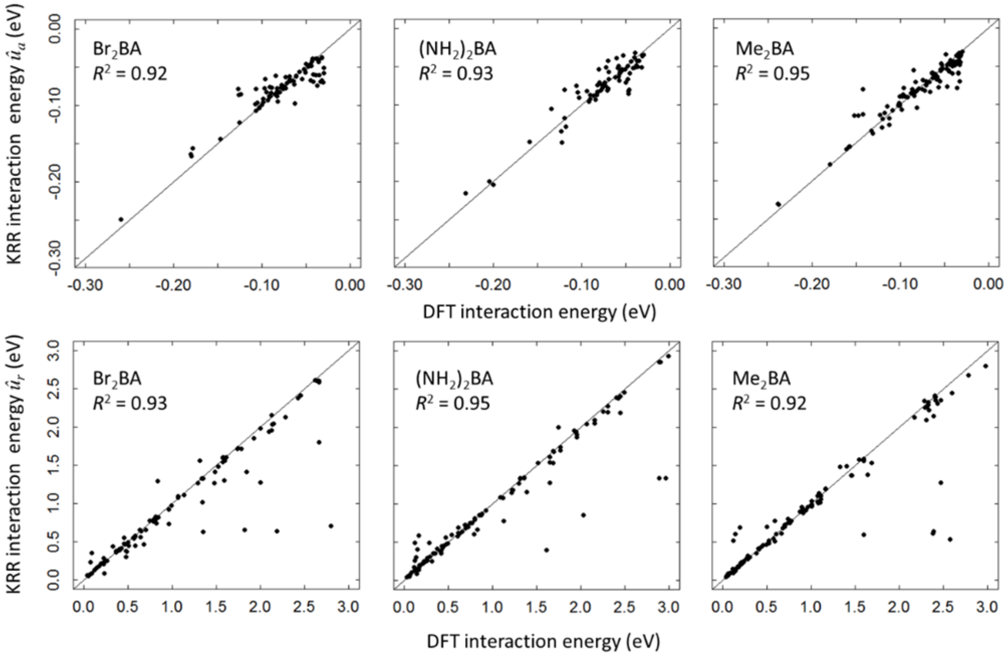

Supplementary Figure 14. Performance of the kernel ridge regression estimators at predicting attractive interaction energies (top row) and repulsive interaction energies (bottom row). Training set size / test set size: 2441 / 129 ($\hat{u}_a$ for Br_2BA), 1083 / 121 ($\hat{u}_r$ for Br_2BA), 1326 / 70 ($\hat{u}_a$ for $(NH_2)_2BA$), 1132 / 126 ($\hat{u}_r$ for $(NH_2)_2BA$), 2058 / 109 ($\hat{u}_a$ for Me_2BA), 1266 / 141 ($\hat{u}_r$ for Me_2BA).

Supplementary Note 5. Equivalence Class Sampling for the GBA Model

This section gives a precise description of the equivalence class sampling (ECS) method. Much of the following material has been presented in a previous paper in the context of the 'chiral block assembly model' (Supplementary Reference 14). Here we present the theory with respect to the GBA model used in this paper. Unless stated otherwise, 'molecules' refers to the abstract molecules in the GBA model, rather than the 'real' molecules from Supplementary Figure 2.

Definitions for the GBA model

We first give a precise description of the GBA model (Supplementary Figure 15). The GBA model is defined with respect to a finite, two-dimensional lattice **L** containing $D = \sqrt{D} \times \sqrt{D}$ points with lattice vectors **a** and **b**, respectively, where D, a, and b are finite. The set of vectors

$$\mathbf{Q} = \{\mathbf{q}_{nm}\}_{n,m=0,1,\ldots,\sqrt{D}}, \tag{10}$$

where $\mathbf{q}_{nm} = n\mathbf{a} + m\mathbf{b}$, is called the *coordinates* of **L**. We define a grid **G** containing cells C_{00}, C_{01}, …, $C_{\sqrt{D}\sqrt{D}}$ in such a way that there is a one-to-one correspondence between the cells of **G** and the lattice points of **L**. This can be achieved by assigning the coordinate $\mathbf{q}_{ij}$ to cell C_{ij}.

In addition to **G**, we consider the sets $R = \{\sigma_1, \sigma_2, \ldots, \sigma_r\}$ and $S = \{\theta_1, \theta_2, \ldots, \theta_s\}$. R is called the *color set* and $\sigma_1, \sigma_2, \ldots, \sigma_r$ are called *colors*. S is called the *shade set* and $\theta_1, \theta_2, \ldots, \theta_s$ are called *shades*. r and s are finite. A subset $S_k = \{\theta_{k(1)}, \theta_{k(2)}, \ldots, \theta_{k(h)}\}$ of the shade set is assigned to color σ_k. A *molecule* is any triple of the form $\{C_{hi}, \sigma_j, \theta_k\}$, where $C_{hi} \in G$, $\sigma_j \in R$, and $\theta_j \in S_j$. The set of all molecules is

$$Z = \bigcup_{C \in G, \sigma_k \in R} \left(\bigcup_{\theta \in S_k} \{C, \sigma_k, \theta\} \right). \tag{11}$$

Let N be a given and finite integer. The *configuration space* Ω is the collection of all unordered N-tuples from Z that meet the following condition: each of the N molecules

possesses a unique unit cell. This condition is referred to as the *single occupancy restriction.* The elements c_1, c_2, …, of Ω are called *configurations*. Ω is a finite collection.

Suppose we are given a function $d : Z \times Z \rightarrow \mathbf{R}_+$ which measures the 'distance' between molecules. For now, we consider the case where

$$d\left(z_i, z_j\right) = \left|\mathbf{s}_i - \mathbf{s}_j\right| \tag{12}$$

where $\mathbf{s}_i$ and $\mathbf{s}_j$ are the coordinates assigned to the cell belonging to z_i and z_j, respectively, and | | denotes the ordinary Euclidean distance. Let c be a configuration from Ω. A subset of molecules $I = \{z_1, z_2, \ldots z_m\}$ from c is said to be an *island* if and only if

1. For any $z_k \in I$, there is another $z_j \in I$ such that $z_k \neq z_j$ and $d(z_k, z_j) \leq M_c$, where M_c is given parameter called the *cut-off distance*.

2. For any pair z_i and z_j from c such that $z_i \in I$ and $z_j \notin I$, $d(z_i, z_j) > M_c$.

The concept of islands is illustrated in Supplementary Figure 16. Given a configuration c, we can uniquely determine the islands contained in c. By definition, a single molecule z may constitute an island if $d(z, z_j) > M_c$ for all other z_j in c.

Finally, consider the two configurations c_1 and c_2 shown in Supplementary Figure 17. c_1 contains islands I_1 and I_2, and c_2 contains islands J_1 and J_2. I_2 can be mapped onto J_1 by translation across the grid, and I_1 can be mapped onto to J_2 by translation followed by rotation. In general, consider two configurations c_i and c_j, and let W_i and W_j denote the set of islands belonging to c_i and c_j, respectively. If there is a one-to-one and onto transformation $f : W_i \rightarrow W_j$ that maps the islands of c_i onto the islands of c_j by translations and rotations of islands alone, then c_i and c_j are *rotational isomorphs*. We can therefore define a relation ~ on Ω such that $c_i \sim c_j$ if and only if c_i and c_j are rotational isomorphs. ~ is an equivalence relation, and therefore we can partition Ω into equivalence classes h_1, h_2, …, h_m (Supplementary Figure 18). The collection $H = \{h_1, h_2, \ldots, h_m\}$ is called the *reduced configuration space*.

Note that for fixed N and all D greater than some D_0, the equivalence class space H does

not depend on the magnitude of D. Under these conditions, the grid G is large enough to.

accommodate all possible island combinations available for a fixed N. We refer to this as the *H-invariance property*. Throughout the following, we will always assume that $D > D_0$

<u>*Canonical representations*</u>

Equivalence classes from H can be unambiguously identified by means of *canonical representations*. Let h_k be an equivalence class, choose an arbitrary configuration c from h_k, and let $S = \{I_1, I_2, \ldots\}$ be the islands belonging to c. The collection

$$can_k = \{S, \varnothing\}, \tag{13}$$

where Ø denotes the empty set, is called a *canonical representation* of h_k (Figure 19). In general there is more than one canonical representation possible for a given equivalence class (indeed, each configuration contained in the equivalence class yields its own canonical representation). However, all canonical representations available to a given equivalence class are equivalent up to rotational isomorphism f (where we enforce $f(\varnothing) = \varnothing$). By convention, we do not distinguish canonical representations that only differ by rotational isomorphism.

The *islands belonging to* h_k are defined as the set can_k - Ø, where can_k is a canonical representation of h_k. Again, the islands belonging to h_k are unique up to rotational isomorphism.

<u>*Equivalence class sampling*</u>

The purpose of equivalence class sampling is to obtain a sample of equivalence classes with respect to the probability distribution $v : H \to [0, 1]$. This is implemented *via* the Metropolis-Hastings algorithm (Supplementary Reference 15). The Metropolis-Hastings algorithm involves simulating a Metropolis-Hastings Markov chain, defined as follows.

Definition 5.3.1. Generating Chain and Metropolis-Hastings Chain

i. Construct a Markov chain Y on the reduced configuration space H with transition matrix $\mathbf{Q} = [q(h_i, h_j)]_{T \times T}$, where $q(h_i, h_j)$ is the probability for Y to jump from equivalence class h_i to equivalence class h_j in a single step, and T is the number of equivalence classes in the reduced configuration space H. Y is referred to as the *generating chain*.

ii. Define another Markov chain Z on H with transition matrix $\boldsymbol{\alpha} = [\alpha(h_i,h_j)]_{T \times T}$, where

$$\alpha\left(h_i,h_j\right) = q\left(h_i,h_j\right)\min\left(1,\frac{q\left(h_j,h_i\right)v\left(h_j\right)}{q\left(h_i,h_j\right)v\left(h_i\right)}\right) \tag{14}$$

is the probability for Z to jump from equivalence class h_i to equivalence class h_j in a single step. Z is referred to as the *Metropolis-Hastings chain*. Simulation of Z is called the *Metropolis-Hastings algorithm.*

The Metropolis-Hastings chain has the stationary probability distribution v, providing that the *generating chain Y* is v-irreducible. Y is said to be v-irreducible if it has a non-zero probability of moving from h_i to h_j within a finite number of steps whenever $v(h_i) > 0$ and $v(h_j) > 0$.

In this work, a generating chain Y with state space H was constructed with the so-called *extension-reduction transformation*. Two definitions are required in order to introduce this. Consider an arbitrary equivalence class h_k and two arbitrary islands I and J from a canonical representation can_k of h_k.

Definition 5.3.2.

Let I_a be the set of islands that could result from by adding a single molecule to I, i.e.,

$$I_a = \left\{ I \cup z : \min_{z_i \in I} d\left(z_i, z\right) \le M_c, z \in Z \right\}. \tag{15}$$

Now, eliminate elements from I_a until there are no pairs of islands in I_0 which

are rotational isomorphs. Denote the resulting set I_+. I_+ is called the *extension set* of island I. If $I = \emptyset$ (the empty set), then we set $I_+ = Z$.

Definition 5.3.3.

Let J_b be the set of islands that could result from removal of a single molecule from J, i.e.,

$$J_b = \{J - z : z \in J\}. \qquad (16)$$

Now, remove elements from J_b until J_b contains no pairs of islands in J_0 which are rotational isomorphs. Denote the resulting set by J_-. J_- is called the *reduction set* of island J. J_- only exists when $J \neq \emptyset$.

The extension-reduction transformation is defined by the following steps.

Definition 5.3.4. Extension-Reduction Transformation

Step 1. Fix a canonical representation can_k of h_k and choose an island I_i from can_k with probability

$$p_i \propto |I_i|^{\alpha_1}, \qquad (17)$$

where α_1 is a positive constant and $|I_i|$ is the number of molecules in island I_i. Now, choose another island $I_j \neq I_i$ from can_k with probability

$$q_j \propto \left(1 + |I_j|\right)^{\alpha_2} \qquad (18)$$

where α_2 is another positive constant.

Step 2. Replace I_i (respectively I_j) with a randomly chosen element from I_{i+} (respectively I_{j-}), and call the resulting set can_k' The island I_i (respectively I_j) is said to be *extended* (respectively *reduced*).

Step 3. Remove or add empty elements Ø to can_k' so that can_k' contains exactly one empty element. The resulting set is a canonical representation for an equivalence class $h_{k'}$ that may or may not be different from h_k. This step is included to cover the cases where an empty element Ø is extended or an island containing only one molecule is reduced during step 2.

An example of an extension-reduction transformation is shown in Supplementary Figure 20. The generating Markov chain Y for equivalence class sampling is defined as follows.

Definition 5.3.5. Extension-Reduction Chain

Fix an equivalence class h_k and let $ER(h_k)$ denote an extension-reduction transformation on h_k. Set $Y_0 = h_k$, $Y_1 = ER(Y_0)$, $Y_2 = ER(Y_1)$, and so on. The sequence $Y = \{Y_0, Y_1, \ldots\}$ is a Markov chain on H called the *extension-reduction chain*.

A very similar extension-reduction chain to the above has been analyzed in detail previously (Supplementary Reference 14). Following the proof of Theorem 2 from that paper, we can show that the extension-reduction chain defined above is v-irreducible. Moreover, by a straightforward application of the technique presented there (Appendix v), we can compute the transition probabilities $q(h_i, h_j)$ for simulating the Metropolis-Hastings chain.

<u>*The degeneracy factor and low-coverage approximation*</u>

In this paper, the specific probability distribution considered is of the form

$$v(h_k) = \frac{1}{Q} n_k e^{-\varepsilon(c \in h_k)/k_B T} \qquad (19)$$

where the 'degeneracy factor' n_k is the number of configurations contained in equivalence class h_k, k_B is the Boltzmann constant, T is the temperature, and $\varepsilon(c \in h_k)$ is the energy for any configuration c contained in equivalence class h_k. We assume that $\varepsilon(c \in h_k)$ is independent of the choice of configuration c contained in equivalence class h_k.

The partition function Q in Supplementary Equation (19) is defined as

$$Q = \sum_{h_k \in H} n_k \exp\left(-\frac{\varepsilon\left(c \in h_k\right)}{k_B T} \right) \tag{20}$$

To perform equivalence class sampling, the degeneracy factors in Supplementary Equation (19) for each equivalence class in H must be known *a priori*. Unfortunately, it is not possible to calculate n_k exactly in the general case. The calculations presented in the main paper therefore employ a *low-coverage approximation*. To define this approximation, several definitions are needed.

Definition 5.4.1.

Fix an arbitrary equivalence class h_k. For an arbitrary island I from the set of islands belonging to h_k, define the set

$$r\left(I\right) = \left\{ z : \min_{z_i \in I} d(z_i, z) \le M_c, z \notin I, z \in Z \right\}. \tag{21}$$

$r(I)$ is the set of molecules that cannot occur when island I is present on the grid. The *exclusion area* of I is the set $R(I) = I \cup r(I)$.

Definition 5.4.2.

The *coordinates assigned to molecule* $z = \{C_{hi}, \sigma_j, \theta_k\}$ are the coordinates from $\mathbf{L}$ associated with cell C_{hi}. For an arbitrary island I containing w molecules, let $\mathbf{s}_1, \mathbf{s}_2, \ldots, \mathbf{s}_w$ be the coordinates assigned to the molecules $z_1, z_2, \ldots, z_w$ of I. Let

$$\boldsymbol{\rho}\left(I\right) = \frac{1}{w} \sum_{j=1}^{w} \mathbf{s}_j \,. \tag{22}$$

The *center of mass* of I, denoted $R^*(I)$, is defined as

$$R^*\left(I\right) = \arg\min_{z_k \in I} \left\{ \left| \mathbf{s}_k - \rho\left(I\right) \right| \right\}, \tag{23}$$

Without loss of generality, we assume that $R^*(I)$ is unique for a given island I.

Definition 5.4.3. Equivalence class degeneracy and pseudo-degeneracy

Fix a canonical representation can_k for h_k and define the numbers

n_k = number of ways of placing the islands in can_k on the grid *via* translations and rotations such that, for any two islands I_k, I_j, we have $R(I_k) \cap I_j = R(I_j) \cap I_k = \emptyset$.

(24)

n_k^* = number of ways of placing the islands in can_k on the grid *via* translations and rotations such that, for any two islands I_k, I_j, we have $R^*(I_k) \cap R^*(I_j) = \emptyset$.

(25)

The number n_k^* counts the number of configurations without regard for exclusion areas. Consequently, we have $n_k < n_k^*$ for any equivalence class k. n_k and n_k^* are called the *degeneracy factor* and the *pseudo-degeneracy factor* for equivalence class h_k, respectively.

Definition 5.4.4. Low-coverage approximation

The *low coverage approximation* is where we write

$$n_k \approx n_k^* \tag{26}$$

i.e., where we replace n_k in Supplementary Equation (19) with the number n_k^* when carrying out equivalence class sampling.

Unlike n_k, we can derive an exact formula for the pseudo-degeneracy n_k^*. This formula is a generalization of the formulas 5 and 6 in the main text to equivalence classes containing arbitrary numbers of islands. Again, several additional concepts must be

defined to proceed.

Definition 5.4.5. Translational isomorphs

> Fix an equivalence class h_k and choose two configurations c_1 and c_2 from h_k. Let S_1 and S_2 be the set of islands belonging to c_1 and c_2, respectively. We say that c_1 and c_2 are *translational isomorphs* if there is a one-to-one and onto map $g : S_1 \rightarrow S_2$ that transforms the islands in S_1 into the islands of S_2 by *translations* alone.

Note that if c_1 and c_2 are translational isomorphs then they are also rotational isomorphs. However, the converse is not necessarily true.

Definition 5.4.6. Subclasses

> Define a relation $\sim^*$ on h_k such that $c_i \sim^* c_j$ if and only if c_i and c_j are translational isomorphs. $\sim^*$ is an equivalence relation, and under $\sim^*$ we can partition the equivalence class h_k into equivalence classes g_{k1}, g_{k2}, … (Supplementary Figure 21). g_{k1}, g_{k2}, … are referred to as *subclasses* of equivalence class h_k.

As with equivalence classes, we can represent subclasses using canonical representations. Fix an equivalence class h_k and subclass g_{kj}. A canonical representation can_{kj} for g_{kj} can be obtained by choosing any configuration c belonging to g_{kj} and letting can_{kj} to be the set of islands belonging to c and one empty element. As before, the various canonical representations possible for a given subclass are equivalent up to translational isomorphism (where we enforce $g(\varnothing) = \varnothing$ as before).

Definition 5.4.7. Translational Classes

> Fix h_k and let can_{kj} be canonical representational for subclass g_{kj}. Let $\sim^{**}$ be a relation on can_{kj} such for any two islands $I, J \in \text{can}_{kj}$, $I \sim^{**} J$ if any only if I can be superimposed onto J *via* translations alone (where we enforce $\varnothing \sim^{**} \varnothing$). $\sim^{**}$ is again an equivalence relation, and under $\sim^{**}$ can_{kj} is partitioned into equivalence classes a_{kj1}, $a_{kj2,}$ … . a_{kj1}, $a_{kj2,}$ are referred to as *translational classes*.

Translational classes are illustrated in Supplementary Figure 22. Finally, fix an equivalence class h_k. We can write

$$n_k^* = n_{k1}^* + n_{k2}^* + \cdots + n_{kr}^* , \tag{27}$$

where

n_{kj}* = number of ways of placing the islands of can_{kj} on the grid *via* translations such that, for any two islands I_k, I_j, we have $R^*(I_k) \cap R^*(I_j) = Ø$.

Now, fix a subclass g_{kj} and canonical representation can_{kj}. Let A_{kj1} be the number of islands from can_{kj} belonging to translational class a_{kj1}, A_{kj2} be the number of islands belonging to translational class a_{kj2}, and so on. We can write

$$n_{kj}* = \begin{pmatrix} D \\ D - m_k, A_{kj1}, A_{kj2}, \ldots \end{pmatrix}, \tag{28}$$

where $m_k = A_{kj1} + A_{kj2} + \cdots$ is the number of islands contained in can_{kj}. Supplementary Equation (28) is the number of ways of placing the islands of can_{kj} on the grid G, such that each island only occupies a single cell of the grid, and is a generalization of the formula 5 from the main text. By substituting Supplementary Equation (27) into (26), we arrive at the generalization of formula 6 from the main text, namely

$$n_k* = \sum_{TC_k} \begin{pmatrix} D \\ D - m_k, A_{kj1}, A_{kj2}, \ldots \end{pmatrix}, \tag{29}$$

where the subscript TC_k means that the sum runs over all translational classes of can_{kj}. Supplementary Equation (29) is exact within the low-coverage approximation.

Justification of Supplementary Equation (26) under the low-coverage approximation

The low-coverage approximation (Supplementary Equation (26)) is accurate when the

size of the grid D is very large compared to the number of molecules N. Indeed, let D be the number of cells in the GBA model and suppose that $D > D_0$. Under this condition, the GBA model satisfies the H-invariance condition, and it is possible to hold an equivalence class h_k fixed while increasing D. Letting n_k and n_k* be the degeneracy and pseudo-degeneracy of an equivalence class h_k, we can prove that

$$n_k / n_k^* \to 1 \tag{30}$$

as $D \to \infty$.

The proof of Supplementary Equation (30) follows almost the same steps as in Appendix IV of Supplementary Reference 14. Suppose that equivalence class h_k contains m islands.

Step 1.

Let **L** be the lattice with respect to which the GBA model is defined. Write **L = X** × **Y**, where **X** = **Y** = {1, 2, …, √D}, and let x and y be any distinct choice of m points from **X** and **Y**, respectively. We call $x \times y$ a *sublattice*. We consider two families of sublattices, F_0 and F_1. Sublattices from F_0 are generated without any restrictions on what points may be chosen from **X** and **Y**. The number of sublattices contained in F_0 is equal to

$$s_0 = \binom{\sqrt{D}}{m}^2 . \tag{31}$$

Sublattices from F_1 are generated in the following way. Fix an integer L and choose m mutually exclusive sets of L consecutive integers $J_1, J_2, \ldots, J_m$ from **X**. Independently of $J_1, J_2, \ldots, J_m$, choose another m mutually exclusive sets of L consecutive integers J_1', J_2', …, J_m' from **Y**. Let

$$x = \bigcup_{k=1}^{m} \min\left(i \in J_k\right) \tag{32}$$

and

$$y = \bigcup_{k=1}^{m} \min\left(i \in J_k'\right). \quad (33)$$

The set $x \times y$ is a sublattice, and F_1 is the union of all such sublattices which can be constructed in this way. Letting P_m be the number of ways to choose m mutually exclusive sets of L consecutive integers $J_1, J_2, \ldots, J_m$ from **X**, the number of sublattices in F_1 is therefore

$$s_1 = P_m^2. \quad (34)$$

Following exactly the procedure in Step 1 from Appendix iv of Supplementary Reference 14 (with the symbol H replaced by L and d replaced with $\sqrt{D}$), we arrive at

$$P_m = \sum_{q=0}^{\sqrt{D}-mL} \binom{q+m-1}{q}. \quad (35)$$

Then, following exactly the procedure in Step 2 from Appendix IV of Supplementary Reference 14, we have that

$$s_1/s_0 \to 1 \quad (36)$$

as $\sqrt{D} \to \infty$.

Step 2.

Fix a canonical representation can_k for equivalence class h_k, and let $I_1, I_2, \ldots, I_m$ denote the islands (excluding the empty island) contained in can_k. For each $1 \leq k \leq m$, choose one molecule z_k' from I_k. z_1', z_2', …, z_m' are called *tagged molecules*. Any configuration belonging to h_k can be generated in the following way. Choose a sublattice from F_0 and identify the corresponding subset of cells **g** in the grid **G** of the GBA model. Let **g**' be any choice of m cells from **g**, and place $I_1, I_2, \ldots, I_m$ on **G** such that the following conditions are satisfied.

(a) The cells of z_1', z_2' …, z_m' are mutually exclusive and are entirely contained in **g**'.

(b) For any two islands I_i and I_j, there is no pair $z_a \in I_i$ and $z_b \in I_j$ such that $d(z_a, z_b) \leq M_c$.

If (b) cannot be fulfilled, we choose another grid from F_0 and start again. There will always be a grid in F_0 which permits (b) providing that D is large enough. We call the above procedure T. We also define another procedure T^*, which differs from T only in that (b) is not enforced. Furthermore, we define another procedure T_1, which is identical to T^* except that F_0 is replaced with F_1.
Now, any configuration in h_k can be generated by procedure T. The number of unique configurations that T can yield (up to rotational isomorphism) is n_k, the degeneracy of h_k. Similarly, the number of unique configurations that T^* can yield is equal to the pseudo-degeneracy n_k^*. Let n_k^1 be the number of unique configurations that procedure T_1 can yield. For sufficiently large L and $\sqrt{D} > mL$, we therefore have

$$n_k^1 < n_k < n_k^* \tag{37}$$

The proof of Supplementary Equation (30) will be complete if we can show that $s_1/s_0 \rightarrow 1$ implies $n_k^1/n_k^* \rightarrow 1$. To do this, observe that the family F_1 is a subset of F_0. We can therefore write the identity

$$F_1 = F_0 - \left(F_0 - F_1\right). \tag{38}$$

$s_1/s_0 \rightarrow 1$ then implies that $F_0 - F_1 \rightarrow \varnothing$ as $\sqrt{D} \rightarrow \infty$. However, according to the constructions of T^* and T_1, the numbers n_k^1 and n_k^* only differ when F_1 and F_0 contain different grids. It therefore follows that $n_k^1/n_k^* \rightarrow 1$, and hence that $n_k^*/n_k \rightarrow 1$.

Status of the low-coverage approximation under higher coverage conditions

According to the inequality in Supplementary Equation (37), for any sufficiently large $\sqrt{D}$, there exists a constant A such that

$$n_k^1 / n_k^* < A < 1, \tag{39}$$

$$A \to 1 \text{ as } \sqrt{D} \to \infty, \tag{40}$$

and

$$n_k = A n_k^* . \tag{41}$$

The configurational entropy $S = k_B \ln n_k$ can therefore be written as

$$S = S_0 + \delta S , \tag{42}$$

where $S_0 = k_B \ln n_{k^*}$ is the entropy computed under the low coverage approximation, and $\delta S_0 = k_B \ln A$ is a negative-valued 'correction term'. Supplementary Equation (41) demonstrates that the low-coverage approximation is accurate up to a factor ln A, which is very small for large $\sqrt{D}$. Unfortunately, A is very difficult to examine numerically, due to the very large values that n_k^1 and n_k* take as D becomes large.

Under 'intermediate coverage' conditions, under which the H-invariance condition holds (see the end of section 5.1), but A is appreciably different than 1, the effect of entropy on the molecular self-assembly process will therefore have contributions from both the term S_0 and the correction term δS_0. While the calculations presented here will not capture the effect of δS_0 under intermediate coverage conditions, they will still accurately describe the effect of S_0. This is likely the reason why our theory can explain aspects of the STM images shown in Figures 4 and 7 of the main paper.

<u>*Computational Details*</u>

In section 4.1, the Euclidean distance was used to measure the distance $d(z_i, z_m)$ between molecules. However, for the actual simulations presented in this paper, a more realistic metric was employ, as follows. Here, 'physical molecules' mean the molecule conformations shown in Supplementary Figure 1, in contrast to 'molecules' which refer to abstract molecules in the GBA model.

1. Supplementary Figure 6 plots the locations of the adsorption sites in the Cu(111) unit cell, as identified by density functional theory. The locations of these adsorption

sites are given by the vectors $\mathbf{r}_5$, $\mathbf{r}_6$…, $\mathbf{r}_{25}$, where the indices 5, 6, …, 25 correspond to the labels of the adsorption sites in Supplementary Figure 6. These adsorption sites correspond to the colours in the GBA model. Suppose that $z_i = (C_{jk}, \sigma_h, \theta_l)$, and define the vector

$$\mathbf{R}_i = \mathbf{q}_{jk} + \mathbf{r}_{n(h)} - \mathbf{r}_7 \tag{43}$$

where $\mathbf{q}_{jk}$ is the coordinate assigned to cell C_{jk} (see section 5.1) and $n(h)$ is the label of the adsorption site in Supplementary Figure 6 that corresponds to the color σ_h. A physical molecule with the conformation given in Supplementary Figure 2 was generated such that its center of mass lies directly above the point $\mathbf{R}_i$ and its orientation is given by θ_l.

2. Another physical molecule was generated according to the above procedure for the molecule z_m. The distance $d(z_i, z_m)$ was then defined as the shortest atom-atom distance between the physical molecule generated from z_i and the physical molecule generated from z_m.

In this study, the cut-off distance M_c was set to 8 Å, and the lattice vectors **a** and **b** set to (2.52 Å, 0) and (-1.26 Å, 2.18 Å) respectively, which corresponds to the lattice constants for the Cu(111) unit cell.

In this study, equivalence class sampling was implemented as above using the energy function constructed in Section 2 and 3, the low coverage approximation, D = 2500 cells, and extension-reduction chain parameters $\alpha_1 = \alpha_2 = 0$. The Metropolis-Hastings algorithm was supplemented with the parallel tempering algorithm described in Supplementary Reference 16) in order to improve convergence rates. For parallel tempering, 11 replicas at temperatures 200 K, 210 K, …, 290 K and 300 L were used. Our code utilized routines from R and the packages igraph and FNN (Supplementary References 6, 17, 18). Simulations for the $(Br)_2BA$ system were ran for 1,200,000 steps and appear to converge after about 800,000 steps. Simulations for the $(NH_2)_2BA$ and $(CH_3)_2BA$ system were ran for 900,000 and 600,000 steps, respectively, and appeared to converge after about 300,000 steps. This fast convergence time is a due to the relatively small size of the reduced configuration space H. 100 island combinations (equivalence classes) randomly chosen from the final 300,000 simulation steps were used for analysis

in each case. The assembly probability for chain-shaped islands, amorphous islands, and single molecules was estimated by

$$p_x = \frac{1}{100}\sum_{k=1}^{100}\left(\frac{n_{xk}}{n_k}\right) \tag{44}$$

where p_x is the assembly probability for x-type islands (x = chain-shaped, amorphous, single molecule), n_k is the number of islands in island combination k, and n_{xk} is the number of x-type islands in island combination k. The numbers n_{xk} were counted by visual inspection of island combinations.

The software VESTA was used to visualize the islands and generate the molecule images used in the figures in this paper (Supplementary Reference 19).

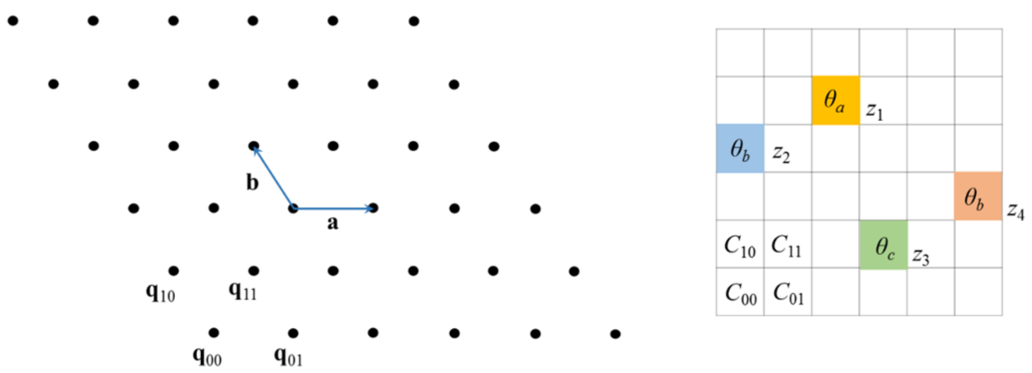

Supplementary Figure 15. Left. Example of a lattice **L**. The lattice vectors **a** and **b** as well as four sites with coordinate vectors $\mathbf{q}_{00}$, $\mathbf{q}_{01}$, $\mathbf{q}_{10}$, and $\mathbf{q}_{11}$ are labelled. Right. Grid corresponding to **L**. The cells labelled C_{00}, C_{10}, C_{01}, and C_{11} correspond to the lattice sites with coordinate vectors $\mathbf{q}_{00}$, $\mathbf{q}_{01}$, $\mathbf{q}_{10}$, and $\mathbf{q}_{11}$, respectively. Four molecules z_1, z_2, z_3 and z_4 placed on the lattice are shown. In this case, $\sqrt{D} = 6$.

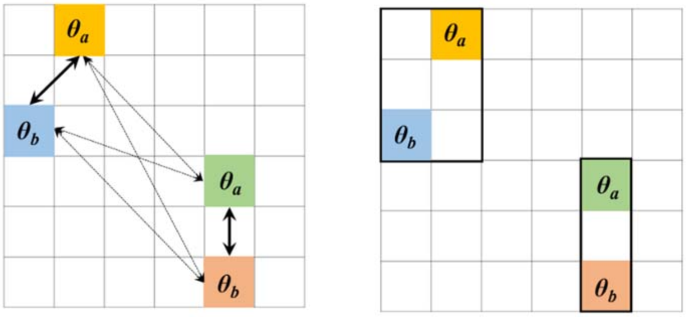

Supplementary Figure 16. (Left) One configuration of the GBA model with four molecules. Thick (thin) black arrows represent pairs of molecules that are separated by a distance less than (greater than) M_c under the metric d. (Right) The black rectangles represent groups of molecules that are contained within the same island. The black rectangles are only used to indicate the islands, and are not involved in the definition of islands.

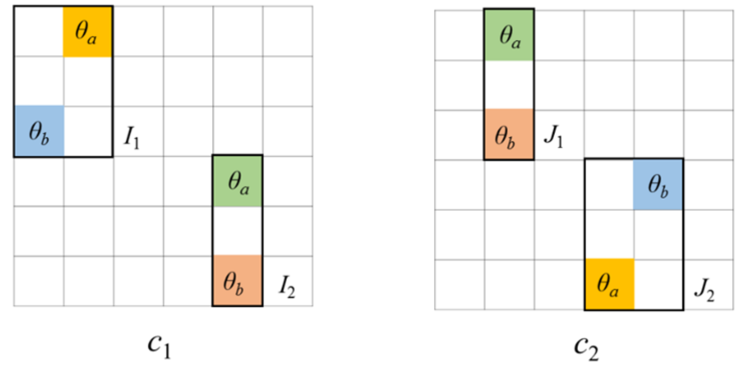

Supplementary Figure 17. Two configurations that are a pair of rotational isomorphs.

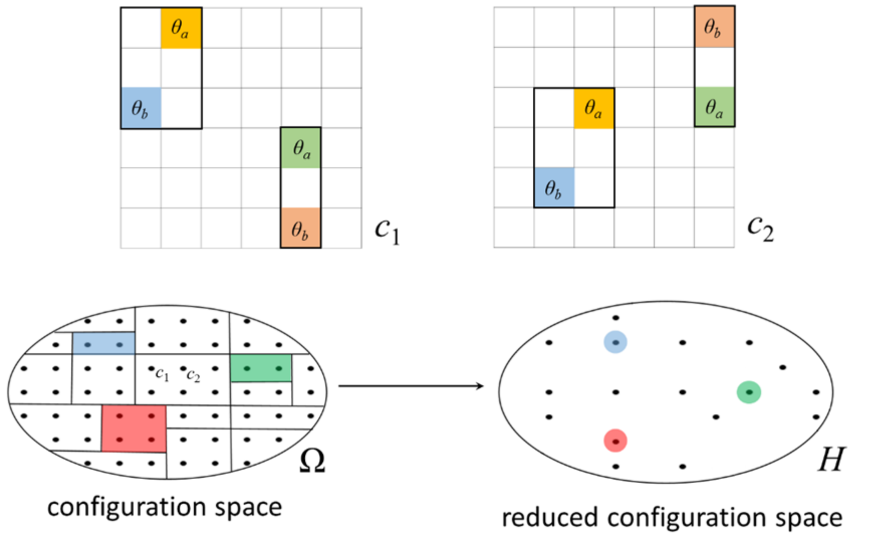

Supplementary Figure 18. Partitioning of the configuration space Ω into equivalence classes. Each point in Ω corresponds to one configuration. The regions of Ω resulting from the partitioning correspond to the equivalence classes, and any configuration contained in one equivalence class can be transformed into any other configuration in the same equivalence class by rotational isomorphism. In the reduced configuration space *H*, each point corresponds to one equivalence class in Ω.

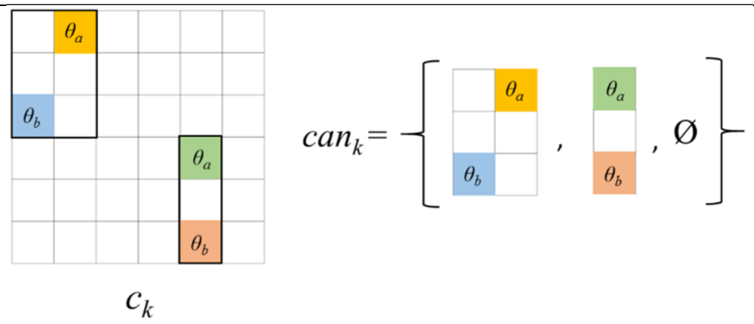

Supplementary Figure 19. A canonical representation for the equivalence class h_k, where configuration c_k belongs to h_k. The islands in c_k are indicated by the black rectangles.

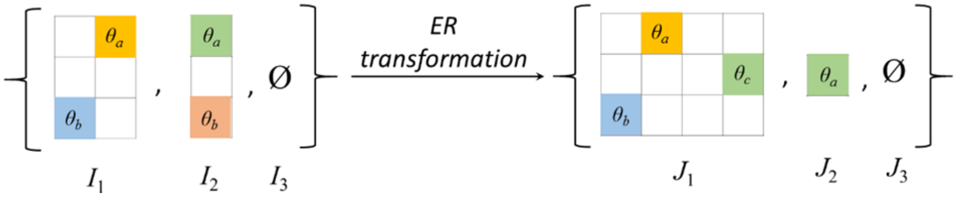

Supplementary Figure 20. Example of an ER (extension-reduction) transformation. In this case, the island I_1 is extended, becoming J_1, and the island I_2 is reduced, becoming J_2.

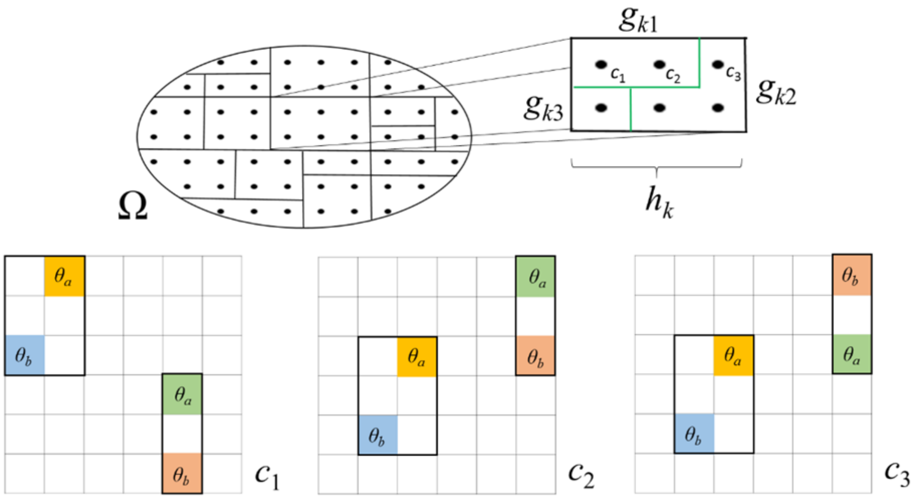

Supplementary Figure 21: Partitioning of an equivalence class h_k into subclasses g_{k1}, g_{k2}, g_{k3}. The configurations c_1, c_2, and c_3 are all rotational isomorphs. However, whereas c_1 and c_2 are translational isomorphs, c_3 is not a translational isomorph of either c_1 or c_2.

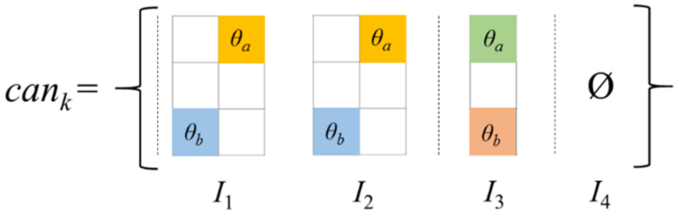

Supplementary Figure 22. Partitioning of the islands of a canonical representation can_k into translational classes. I_1 and I_2 are superimposable upon translation and therefore belong to the same translational class. I_3 and I_4 belong to their own translational class. Consequently, there are three translational classes, and we have $A_{k1} = 2$, $A_{k2} = 1$, and $A_{k3} = 1$.

Supplementary References

1. Kresse, G. & Furthmuller, J. Efficient iterative schemes for *ab initio* total-energy calculations using a plane-wave basis set. *Phys. Rev. B.* **54**, 11169 – 11186 (1996)

2. Perdew, J. P., Burke, K., & Ernzerhof. Generalized Gradient Approximation Made Simple. *Phys. Rev. Lett.* **77**, 3865 - 3870 (1996)

3. Hamada, I. van der Waals density functional made accurate. *Phys. Rev. B.* **89**, 121103 – 121108 (2014).

4. Klimes, J., Bowler, D. R., & Michaelides, A. Van der Waals density functionals applied to solids. *Phys. Rev. B.* **83**, 195131 – 195144 (2011)

5. Klimes, J., Bowler, D. R., & Michaelides, A. Chemical accuracy for the van der Waals density functional. *J. Phys.: Condens. Matter.* **22**, 022201 – 022206 (2010)

6. R Core Team. R: A language and environment for statistical computing. R Foundation for Statistical Computing, Vienna, Austria. https://www.R-project.org/ (2015).

7. Akima, H. & Gabhardt, A. akima: Interpolation of Irregularly and Regularly Spaced Data. R package version 0.12. http://CRAN.R-project.org/package=akima (2015)

8. Bagus, P. S., Hermann, K., & Woll, C. The interaction of C_6H_6 and C_6H_{12} with noble metal surfaces: Electronic level alignment and the origin of the interface dipole. *J. Chem. Phys.* **123**, 183109 – 183123 (2005).

9. Rupp, M. *et al*. Fast and Accurate Modeling of Molecular Atomization Energies with Machine Learning. *Phys. Rev. Lett.* **108**, 058301 – 058306 (2012).

10. Hansen, K. *et al*. Assessment and Validation of Machine Learning Methods for Predicting Molecular Atomization Energies. *J. Chem. Theory Comput.* **9**, 3404 – 3419 (2013).

11. Meyer, D., *et al*. e1071: Misc Functions of the Department of Statistics, Probability Theory Group, TU Wien. R package version 1.6-7.

http://CRAN.R-project.org/package=e1071 (2015)

12. Image of potential curve adapted from http://www.ipme.ru/ipme/labs/dms/prive/ivanova/Home_page_Elena_Ivanova/Moment%20potentials%20ENG.htm. Last accessed on 1 September 2016.

13. Hainmueller, J. & Hazlett, C. KRLS: Kernel-based Regularized Least squares (KRLS). R package version 0.3-7. http://CRAN.R-project.org/package=KRLS (2014)

14. Packwood, D. M., Han, P., & Hitosugi, T. State Space Reduction and Equivalence Class Sampling of a Molecular Self-Assembly Model. *Roy. Soc. Open Sci.* **3**, 150681 – 15701 (2016).

15. Robert, C. L. & Casella, G. *Monte Carlo Statistical Methods*. Springer, New York (2004)

16. Geyer, C. J. Importance Sampling, Simulated Tempering, and Umbrella Sampling. In *Handbook of Markov Chain Monte Carlo* (Ed. Brooks, S., Gelman, A., Jones, G. L., Meng, X.L.) 299 - 300. CRC Press, Boca Raton, Florida (2011)

17. Csardi, G. & Nepusz, T. The igraph software package for complex network research. *InterJournal Complex Systems* (http://interjournal.org/manuscript_abstract.php?361100992) (2006)

18. Beygelzimer, A. *et al*. FNN: Fast Nearest Neighbor Search Algorithms and Applications. R package version 1.1. http://CRAN.R-project.org/package=FNN (2013).

19. Momma, K., & Izumi, F. VESTA 3 for three-dimensional visualization of crystal, volumetric and morphology data. *J. Appl. Crystallogr*. **44**, 1272 - 1276 (2011).
